# Changes in eating habits and food preferences in breast cancer patients undergoing adjuvant chemotherapy

**DOI:** 10.1038/s41598-021-92138-7

**Published:** 2021-06-21

**Authors:** Rebecca Pedersini, Pierluigi di Mauro, Sara Bosio, Barbara Zanini, Alessandra Zanini, Vito Amoroso, Antonella Turla, Lucia Vassalli, Mara Ardine, Sara Monteverdi, Manuel Zamparini, Cristina Gurizzan, Deborah Cosentini, Chiara Ricci, Edda Lucia Simoncini, Alfredo Berruti

**Affiliations:** 1 Medical Oncology Department, ASST-Spedali Civili of Brescia, Piazzale Spedali Civili 1, 20123 Brescia, Italy; 2 SSVD Breast Unit, ASST-Spedali Civili of Brescia, Brescia, Italy; 3 Department of Clinical and Experimental Sciences, University of Brescia, Brescia, Italy; 4 SSVD Gastroenterology, ASST-Spedali Civili of Brescia, Brescia, Italy

**Keywords:** Medical research, Oncology

## Abstract

Change in eating habits in early breast cancer (EBC) patients during chemotherapy has been poorly studied in the literature. The primary aim of this study was to prospectively evaluate food preferences and weight change in EBC patients before and after adjuvant chemotherapy. From April 2014 to June 2018, 205 EBC patients underwent a dietary assessment according to the following timeline: baseline evaluation (one week before starting chemotherapy, T0); first follow-up (approximately 2–3 months after starting chemotherapy, T1); final follow-up (one week after chemotherapy end, T2). A statistically significant reduction of the following foods was reported after the start of chemotherapy: pasta or rice, bread, breadsticks/crackers, red meat, fat and lean salami, fresh and aged cheese, milk, yogurt, added sugar, soft drinks, alcoholic beverages (wine, beer, and schnapps), and condiments (oil and butter). Conversely, fruit consumption consistently increased. As a result of these changes, a Healthy Eating Index (HEI) specifically developed for this study and suggestive of a balanced diet, significantly increased. Body weight did not increase, despite reduction in physical activity. This prospective study shows that EBC patients tend to adopt “healthier dietary patterns” during adjuvant chemotherapy, leading to a non-change in weight, despite reduction in physical activity.

## Introduction

Breast cancer is the most common cancer and the leading cause of cancer-related death in women worldwide^1^. The widespread use of adjuvant therapies, however, has led to a progressive decline in breast-cancer mortality^2^ and to an increasing proportion of long-term survivors^3^.

Several reports suggested that 50–90% of early breast cancer (EBC) patients reported a significant weight gain in the months and years after diagnosis^4,5^. Adjuvant chemotherapy was found to be an independent predictive factor of weight gain in EBC patients^6–12^. A recent meta-analysis showed, in fact, that chemotherapy leads to a body weight increase of 2.7 kg on average (95% Confidence Interval [CI] 2.0–7.5 kg), with a significant degree of variation due to high heterogeneity between studies^13^. The pathophysiology of weight gain during chemotherapy has not been fully elucidated and is believed to have a multifactorial etiology: among these potential contributors there are premenopausal status at breast cancer diagnosis^14^, fatigue and reduced physical activity^17–19^ along with the consequent reduction in lean body mass and resting energy expenditure^17,18^, increased food intake due to treatment-related appetite^19^, common use of steroids^20^ and anti-depressant drugs^21^ during chemotherapy.

Change in eating habits in EBC patients during chemotherapy with regard to weight gain has been poorly studied in the literature and controversial findings have been reported^25–28^. Therefore, we conducted a prospective study aimed to evaluate food preferences and consumption before, during, and after adjuvant chemotherapy in a series of EBC patients.

## Patients and methods

This trial is a part of “CHANGE” study, a prospective longitudinal single-center trial conducted at the Medical Oncology Unit and Breast Unit of Azienda Socio Sanitaria Territoriale Spedali Civili of Brescia (Italy). This trial is registered in the *ClinicalTrials.gov* database (NCT identification number: NCT03210441) and approved by the local Ethic Committee of Azienda Socio Sanitaria Territoriale Spedali Civili Spedali Civili of Brescia. Written informed consent was obtained from all participants included in the study. The primary aim of this study was to evaluate eating habits changes during adjuvant chemotherapy and up to 12 months after its completion. The secondary aims were to assess weight changes, taste alterations, and other treatment-related side effects during adjuvant chemotherapy and up to 12 months of follow-up after its completion. In this paper, we would like to report the results regarding the eating habit changes observed before, during, and after adjuvant chemotherapy and their relationship with weight and body mass index (BMI) variations.

From April 2014 to June 2018 two hundred and five EBC patients were enrolled in this study. The inclusion criteria were the following: histologically confirmed EBC; eligibility for (neo)adjuvant chemotherapy, regardless of tumor biology and menopausal status; willingness to comply with the study procedures. Patients with poor performance status, poor compliance, advanced cancer disease or previously treated with chemotherapy for other tumors were excluded. Adjuvant chemotherapy regimens were prescribed according to the national and international guidelines (ESMO^23^ e AIOM^24^).

Dietary assessment was conducted by a trained dietician through a questionnaire specifically developed for this study according to the following timeline (Figure 1): baseline evaluation, one week before starting chemotherapy (T0); first follow-up visit, approximately 2–3 months after starting chemotherapy (T1); final follow-up visit, one week after the end of chemotherapy (T2).

Patients were asked to report the consumption frequency and the serving size of the following foods: fruit, vegetables, pasta or rice, bread, breadsticks/crackers, potatoes, white meat, red meat, fish, fat salami, lean salami, eggs, fresh cheese, aged cheese, legumes, milk, yogurt, ice creams, snacks (belonging to sweet or salty ultra-processed foods), added sugar (to coffee, tea, or other hot or cold beverages), soft drinks, wine, beer, schnapps, butter, and oil. The frequency scale was selected according to the Nurses’ Health Study Dietary Questionnaire^25^. In order to accurately estimate the usual serving size of each food category, food photographs of standard portions of the common Italian foods were used^26^. According to the reported frequencies and serving size of each food category, an estimation in grams or milliliters per week was computed through the table of the standard portions proposed by the Italian Society of Human Nutrition^27^; besides, a further estimation of macronutrient intakes was determined using *Microdiet Software* (Downlee System Ltd. Chapel-en-le-Frith, High Peak, UK), selecting the Italian Food Composition Database.

Furthermore, we specifically developed a Healthy Eating Index (HEI) for this study: we considered 14 different food categories and assigned scores from 0 to 1 or from 0 to 2, according to their frequency consumption. The values used for determining the HEI were attributed considering the WCRF recommendations^28^, the Mediterranean Diet frequency foods intake^29^, and the recent Italian food-based dietary guidelines^30^. The HEI ranges from 0 to 24, where a higher score is suggestive of a healthy and well-balanced diet. Further details about the HEI score assignment are reporter in table S1 of supplementary material.

At every time point weight and height were recorded, BMI was calculated and general information about employment, physical activity (defined as at least 150 min per week of moderate physical exercise) and alcohol consumption were gathered. Lastly, the dietician collected these data without providing any recommendation on healthy diet or lifestyle.

### Ethical approval

All procedures performed in this study involving human participants were in accordance with the ethical standards of the institutional and/or national research committee and with the 1964 Helsinki declaration and its later amendments or comparable ethical standards. Informed consent was obtained from all individual participants included in the study.

## Statistical methods

This study aimed to evaluate food habits changes in EBC patients during adjuvant chemotherapy, which could potentially be related to weight gain. The sample size was calculated assuming a proportion (p_0_) of patients reporting weight gain of 50%^5^, and testing the non-inferiority of the study group with a maximum difference (p_1_–p_0_) clinically not relevant to conclude that p_1_ is non inferior to p_0_ equal to 10% (H_0_: p_1_–p_0_ ≤ − 10% vs H_1_: p_1_–p_0_ > − 10%). Considering a type I error of 5% and a power of 85%, the number of patients expected to be included in the study were 177; therefore, assuming 5% of dropouts, we planned to enroll 204 EBC patients.

Categorical variables were expressed as frequencies and percentages; population characteristics were described as mean and relative 95% CI or percentages of patients; the distribution of continuous variables was calculated as mean ± 95% CI. All variables were treated as they were non-normal in distribution, hence only non-parametric tests were used. Friedman test was used to evaluate statistically significant differences between the mean variations of dietary habits, kilocalories, weight, and BMI during chemotherapy across the different time points. Cochran’s Q test for dependent categorical variables was used to evaluate the statistical significance of the variations of lifestyle parameters, such as employment, and physical activity.

Body weight change was defined as the difference in body weight between day 1 of the first chemotherapy cycle and the day 1 of the last cycle. A weight gain or loss ≥ 5% following adjuvant chemotherapy was considered to be clinically meaningful^31,32^: based on this, patients were classified as increased weight or decreased weight, respectively, and as stable if the weight change did not exceed the threshold value of ± 5%. Thus, we also explored the dietary habits in the three weight categories (weight decrease, stable weight, and weight increase); significant differences between T0 to T2 were tested using the Wilcoxon test, while the Kruskal Wallis test was used to evaluate potential differences between the weight categories.

### Results

#### Patients’ characteristics

Characteristics of the two hundred and five EBC patients enrolled in the CHANGE study are listed in Table 1. Median age was 54 years (range 25–80 years); most patients were postmenopausal and had no axillary involvement; furthermore, most tumors were high grade (G3 in 82%) and were positive for estrogen and progesterone receptor in 68.3% and 56% respectively; human epidermal growth factor receptor 2 (HER2) was expressed in 38% of the tumors. The chemotherapy regimen most commonly used was the sequential regimen with anthracyclines and taxanes. All premenopausal patients (40%) reported chemotherapy-induced amenorrhea at the end of the adjuvant treatment.

**Table 1:** Clinical characteristics of the patients at baseline.

Characteristic	Number of patients (%) (n = 205)
Median age (range)	54 (25–80)
**Menopausal status**	
Premenopausal	82 (40%)
Postmenopausal	123 (60%)
**pT**	
1	103 (50.2%)
≥ 2	102 (49.8%)
**pN**	
0	114 (55.6%)
≥ 1	91 (44.4%)
**Histological type**	
No Special Type (NST)	187 (91.3%)
Others	18 (8.7%)
**Estrogen receptor**	
Positive	140 (68.3%)
Negative	65 (31.7%)
**Progesterone receptor**	
Positive	115 (56%)
Negative	90 (44%)
**Grading**	
G1 or G2	20 (9.7%)
G3	168 (82%)
Unknown	17 (8.3%)
**Ki-67 labeling index**	
< 20%	30 (14.6%)
≥ 20%	169 (82.4%)
Unknown	6 (3%)
**HER2**	
Positive	78 (38%)
Negative	127 (62%)
**Chemotherapy regimen**	
Anthracyclines + taxanes (± trastuzumab)	127 (62%)
Anthracyclines	40 (19.6%)
Taxanes (± trastuzumab)	19 (9.2%)
Others	19 (9.2%)

pT: pathological tumor stage; pN: pathological nodal stage; G1: well differentiated tumor; G2 moderately differentiated tumor; G3: undifferentiated tumor; HER2: Human epidermal growth factor receptor 2.

#### Food habits, calories intake and HEI variations during chemotherapy

The estimated amount of foods, beverages, and condiments consumed at each time point (T0, T1, and T2), expressed as grams or milliliters per week, is reported in Table 2. During the course of adjuvant chemotherapy a statistically significant progressive reduction from baseline of the following foods was detected: pasta or rice (p = 0.009), bread (p < 0.001), breadsticks/crackers (p < 0.001), red meat (p < 0.001), lean and fat salami (p < 0.001 each), fresh cheese (p = 0.039), aged cheese (p = 0.011), milk (p = 0.03), yogurt (p = 0.022), added sugar (p < 0.001), soft drinks (p = 0.003), alcoholic beverages (wine, beer, and schnapps, each with p < 0.001), and condiments, such as oil (p = 0.029) and butter (p = 0.014). Conversely, fruit consumption statistically increased (p < 0.001). The consumption of the remaining foods, such as vegetables, potatoes, white meat, fish, eggs, legumes, ice-creams, and snacks did not statistically change.

**Table 2:** Food, beverages, and condiments consumption before, during and after chemotherapy.

Food or beverage	Baseline (T0)	First follow-up (T1)	Final follow-up (T2)	p*
Fruit	1829 (1662–1996)	2165 (1992.7–2337.5)	2249.3 (2077.8–2420.7)	< 0.001
Vegetables	1602.8 (1499.6–1706)	1652.7 (1557.4–1747.9)	1608.6 (1511.9–1705.3)	0.249
Pasta or rice	390.3 (361–418.9)	366 (340.3–391.6)	362.7 (336–389)	0.009
Bread	423.2 (380.5–465.8)	323 (289.2–356.8)	317.9 (285.3–350.5)	< 0.001
Potatoes	260 (230.4–289.6)	255.6.6 (223.2–287.9)	240.5 (212.2–268.7)	0.392
Breadsticks or crackers	104.8 (85.3–124.4)	78.8 (62.7–94.8)	50.7 (38.3–63)	< 0.001
White meat	236.7 (214.5–258.8)	225 (205–245)	227 (207.4–246)	0.487
Red meat	132 (117–148)	102.7 (89.6–115.8)	101.5 (89.7–113.3)	< 0.001
Fish	252 (228–276)	262.6 (236–288.8)	265.2 (239.8–290.6)	0.956
Lean salami	92.8 (80.6–105)	79 (67–91)	61.7 (52.8–70.6)	< 0.001
Fat salami	28 (22–34)	20.3 (14.8–25.9)	14.4 (9.8–19)	< 0.001
Eggs	118.3 (103.8–1132.8)	109.4 (96.2–122.6)	108.3 (97.1–119.5)	0.153
Fresh cheese	195 (169.5–220.5)	165.2 (145.4–185)	158.2 (137.8–178.5)	0.039
Aged cheese	114.3 (97.9–130.8)	95.6 (82.8–108.4)	87.4 (74.8–100)	0.011
Legumes	199.8 (171.7–228)	214.8 (185.8–243.9)	196.3 (168–224.5)	0.592
Milk	448.3 (358–538.5)	362.8 (283.6–441.9)	376.9 (300–453)	0.030
Yogurt	311.3 (237–385.5)	244.5 (194.2–294.8)	225.3 (176–274.5)	0.022
Sugar	75.4 (62.9–87.8)	57.7 (46.5–69)	55.6 (45.4–65.8)	< 0.001
Ice-creams	78.4 (60.5–96.3)	78.3 (61.5–95.7)	70.4 (53.7–87)	0.105
Snacks	170.2 (136–204.4)	131.3 (103.4–159)	143.5 (114.4–172.7)	0.133
Soft drinks	225.7 (153.9–297.4)	180 (123.2–236.8)	138.8 (92.5–185)	0.003
Wine	281.4 (218–344.7)	166 (112.8–219.2)	139.6 (100.3–178.9)	< 0.001
Beer	128.4 (84.2–172.5)	66.4 (35.5–97.3)	62.7 (35–90.5)	< 0.001
Schnapps	2.93 (0.81–5)	1.12 (0.39–2.63)	0.83 (0.53–2.8)	< 0.001
Oil	246 (233.6–258.4)	235 (225.8–246)	231 (221–241.8)	0.029
Butter	18.6 (14.4–22.8)	15.7 (11.9–19.4)	14.4 (10.7–18.2)	0.014

All measures are expressed as mean of grams per week or milliliters per week with corresponding 95% CI in brackets.

*Friedman’s test.

As reported in Table 3, the changes in food preferences translated in a significantly lower kilocalories intake from baseline to the final follow-up visit. As concerns the single macronutrients, EBC patients significantly decreased the consumption of proteins, carbohydrates, and fat (either expressed in grams/day or in kilocalories/day) at the end of the adjuvant treatment. Finally, the HEI significantly changed from T0 to T2, increasing from a median value of 15.4–16.03 (p < 0.001).

**Table 3:** Calories intake, macronutrients, and HEI before and after chemotherapy.

Daily dietary intake	Mean (IC 95%) at baseline (T0)	Mean (IC 95%) at final follow-up (T2)	p*
Total energy (kcal)	1733.39 (1666.38–1800.39)	1544.92 (1499.79–1590.04)	< 0.001
Proteins (g)	61.78 (59.38–64.18)	54.825 (63.02–56.61)	< 0.001
Proteins (kcal)	247.14 (237.55–256.74)	219.28 (121.11–226.45)	< 0.001
Energy percentage (%)	14.51 (14.17–14.85)	14.28 (13.98–14.57)	0.161
Carbohydrates (g)	180.96 (171.89–180.03)	155.96 (149.72–162.20)	< 0.001
Carbohydrates (kcal)	723.85 (687.56–760.13)	623.86 (598.91–648.80)	< 0.001
Energy percentage (%)	41.29 (00.40–0.42)	40.02 (00.39–0.40)	0.011
Fat (g)	79.96 (76.20–83.71)	69.26 (66.99–71.53)	< 0.001
Fat (kcal)	719.67 (685.88–753.46)	623.42 (602.99–643.86)	< 0.001
Energy percentage (%)	41.49 (40.36–42.61)	40.58 (39.70–41.44)	0.08
HEI (score 0–24)	15,4 (15–15.79)	16,03 (15.66–16.40)	< 0.001

*kcal* kilocalories, *g* grams, *HEI* healthy eating index.

*Wilcoxon test.

#### Weight, BMI, and lifestyle variations

Table 4 shows the variations in weight, BMI, and lifestyle during and after adjuvant chemotherapy. While nearly a quarter of women was not employed at baseline, this proportion increased up to more than a half at the first follow-up visit and at the end of treatment. A progressive decline in women who practiced physical activity from baseline to the final follow-up was also observed (52.7% at T0 vs 43.4% at T2).

**Table 4:** Weight, BMI, and lifestyle variations.

		Baseline (T0)	First follow-up (T1)	Final follow-up (T2)	p*
Weight kg (95% CI)		64.8 (63.1–66.5)	64.8 (63.1–66.5)	65.2 (63.5–66.9)	0.202
BMI kg/m^2^ (95% CI)		24.7 (24.1–25.4)	24.7 (24.1–25.4)	24.9 (24.3–25.5)	0.261
Employment Number of patients	Yes	152 (74.1%)	87 (42.4%)	89 (43.4%)	< 0.0001
	No	53 (25.9%)	118 (57.6%)	116 (56.6%)	
Physical activity Number of patients	Yes	108 (52.7%)	90 (43.9%)	89 (43.4%)	0.036
	No	97 (47.3%)	115 (56.1%)	116 (56.6%)	

*kg* kilograms, *BMI* body mass index.

*Friedman’s and Chi-squared test.

Weight variations were categorized as follows: 27 patients reported weight loss (13.2%), 144 maintained a stable weight (70.2%), 34 reported weight gain (16.6%). In Table S2 of supplementary materials we reported the mean weight and BMI differences between these categories: weight and BMI decreased of – 5.41 kg and – 1.95 kg/m^2^ respectively in the weight loss category, whereas they increased of + 6.47 kg and + 2.24 kg/m^2^ in the weight gain category.

#### Correlations between weight categories, eating habits, calories intake and lifestyle

Baseline weight and BMI of patients who lost weight during treatment were respectively 69.2 kg and 26.6 kg/m^2^, which were higher than those of patients who gained weight (60.2 kg and 22.8 g/m^2^). Patients who maintained a stable weight after chemotherapy had a baseline weight of 65 kg and a BMI of 24.8 kg/m^2^ (Table S2).

Change of food habits across these three weight categories are reported in Table 5. Patients who maintained weight reported a statistically significant reduction from baseline in most foods observed in the general population, such as bread, breadsticks/crackers, red meat, fat and lean salami, fresh and aged cheese, yogurt, added sugar, alcoholic beverages, and condiments. As noted above, fruit consumption statistically increased.

**Table 5:** Change of food habits and HEI score across the three weight categories.

Food or beverage	Decreased weight	Stable weight	Increased weight	p*
**Fruit**				
Baseline	2211.11 (1716.81–2669.37)	1737.50 (1544.80–1927.54)	1914.71 (1513.29–2298.42)	0.276
Final follow-up	2072.22 (1769.59–2372.01)	2305.73 (2083.88–2511.98)	2150.74 (1782.80–2501.19)	0.790
p*	0.754	< 0.001	0.222	
**Vegetables**				
Baseline	1379.26 (1144.69–1613.83)	1609.51 (1485.31–1733.71)	1752.06 (1467.32–2036.79)	0.202
Final follow-up	1540 (1306.92–1773.08)	1584.72 (1469.24–1700.20)	1764.541 (1490.93–2037.90)	0.453
*p*	0.755	0.939	0.236	
**Bread**				
Baseline	487.96 (348.15–635.99)	418.23 (370.49–467.01)	392.65 (306.66–486.01)	0.661
Final follow-up	317.59 (229.65–407.41)	316.67 (277.27–357.28)	323.53 (253.68–395.57)	0.977
*p*	0.025	< 0.001	0.073	
**Breadsticks/crackers**				
Baseline	60.00 (28.89–101.10)	108.33 (83.34–133.23)	125.74 (82.07–172.06)	0.072
Final follow-up	21.11 (8.33–36.67)	56.04 (41.04–73.42)	51.62 (29.12–76.32)	0.263
*p*	0.04	< 0.001	< 0.004	
**Potatoes**				
Baseline	351.85 (250.03–453.67)	244.79 (210.21–279.38)	251.47 (187.55–315.39)	0.088
Final follow-up	281.48 (218.42–344.54)	229.51 (196.87–262.16)	254.41 (163.17–345.66)	0.232
*p*	0.529	0.852	0.209	
**White meat**				
Baseline	263.89 (191.61–336.16)	238.19 (210.88–265.51)	208.82 (168.54–249.11)	0.686
Final follow-up	225 (186.31–263.69)	232.64 (207.88–257.40)	203.68 (158.78–248.57)	0.654
*p*	0.812	0.797	0.254	
**Red meat**				
Baseline	146.30 (117.59–185.19)	128.12 (110.43–147.39)	140.44 (102.21–180.84)	0.255
Final follow-up	113.89 (82.41–152.78)	102.95 (88.20–118.05)	85.29 (65.44–105.15)	0.672
*p*	0.179	0.012	0.04	
**Fish**				
Baseline	272.22 (211.80–332.65)	232.99 (207.50–258.47)	316.91 (231.78–402.05)	0.123
Final follow-up	269.44 (209.96–328.93)	250.35 (223.11–277.59	325 (232.94–417.06)	0.360
*p*	0.187	0.851	0.893	
**Lean salami**				
Baseline	98.89 (68.15–135.90)	90.66 (77.26–105.52)	97.50 (70.60–127.05)	0.508
Final follow-up	67.59 (50.00–86.09)	59.20 (49.58–69.06)	67.79 (45.44–96.17)	0.417
*p*	0.274	< 0.001	0.055	
**Fat salami**				
Baseline	20.37 (10.00–34.26)	26.11 (20.56–32.25)	42.65 (20.89–69.11)	0.477
Final follow-up	12.04 (4.63–21.30)	12.22 (8.13–16.63)	25.74 (10.29–47.65)	0.333
*p*	0.148	< 0.001	0.067	
**Fresh cheese**				
Baseline	218.52 (153.73–288.89)	197.22 (167.54–226.74)	166.91 (116.91–236.01)	0.485
Final follow-up	182.41 (128.70–236.11)	159.55 (135.07–185.07)	133.09 (97.81–170.59)	0.451
*p*	0.206	0.035	0.455	
**Aged cheese**				
Baseline	86.11 (56.30–120.36)	113.96 (93.80–134.58)	138.38 (105.59–176.02)	0.073
Final follow-up	80.37 (49.64–112.22)	87.74 (72.81–103.02)	91.91 (62.06–121.76)	0.966
*p*	0.986	0.029	0.068	
**Eggs**				
Baseline	88.89 (57.94–119.84)	123.78 (105.28–142.29)	118.38 (86.95–149.82)	0.146
Final follow-up	86.11 (58.91–113.31)	111.63 (97.31–125.95)	111.76 (89.53–134)	0.266
*p*	0.185	0.875	0.917	
**Legumes**				
Baseline	197.22 (124.12–270.33)	206.94 (171.04–242.85)	172.06 (117.79–226.33)	0.835
Final follow-up	149.07 (93.03–205.12)	200.69 (165.32–236.07)	215.44 (145.13–285.75)	0.538
*p*	0.378	0.759	0.348	
**Milk**				
Baseline	577.78 (302.42–853.14)	469.10 (354.86–583.34)	257.35 (129.79–384.91)	0.178
Final follow-up	511.11 (289.29–732.94)	372.40 (277.48–467.31)	289.71 (143.21–436.21)	0.630
*p*	0.487	0.083	0.618	
**Yogurt**				
Baseline	368.06 (118.06–833.22)	334.64 (255.23–414.90)	167.28 (97.43–250.00)	0.323
Final follow-up	145.83 (76.39–229.17)	246.96 (189.67–309.46)	196.69 (91.91–343.66)	0.927
*p*	0.382	0.019	0.745	
**Sugar**				
Baseline	60.28 (33.70–86.85)	86.28 (70.40–103.61)	41.40 (23.68–60.36)	0.011
Final follow-up	36.85 (15.57–59.61)	60.40 (48.54–74.11)	50.51 (28.82–75.22)	0.109
*p*	0.015	< 0.001	0.398	
**Biscuits**				
Baseline	116.41 (70.18–162.64)	128.41 (106.58–150.23)	171.50 (113.17–229.83)	0.361
Final follow-up	107.72 (66.56–148.89)	104.81 (86.18–123.43)	134.13 (85.27–183)	0.586
*p*	0.917	0.077	0.117	
**Snacks**				
Baseline	118.89 (46.74–191.04)	181.04 (136.15–225.93)	165 (104.33–225.67)	0.178
Final follow-up	157.78 (78.74–236.81)	142.50 (106.01–178.99)	136.76 (73.79–199.74)	0.630
*p*	0.159	0.181	0.555	
**Wine**				
Baseline	180.56 (56.74–356.33)	298.18 (226.15–380.62)	290.44 (171.90–431.87)	0.136
Final follow-up	119.21 (32.41–234.84)	144.10 (102.00–200.28)	136.95 (63.44–229.76)	0.330
*p*	0.187	< 0.001	0.004	
**Beer**				
Baseline	125.28 (12.22–323.89)	130.05 (79.64–186.76)	123.75 (58.24–211.04)	0.278
Final follow-up	9.17 (0.00–27.50)	71.04 (38.96–112.86)	70.37 (26.69–121.32)	0.095
*p*	0.141	0.001	0.066	
**Schnapps**				
Baseline	1.48 (0.00–4.07)	3.33 (0.77–6.73)	2.35 (0.29–5.29)	0.736
Final follow-up	0.37 (0.00–1.11)	1.11 (0.00–3.19)	0.00 (0.00–0.00)	0.574
*p*	0.18	0.008	0.063	
**Oil**				
Baseline	233.33 (199.63–264.44)	245.24 (231.40–259.58)	259.41 (224.41–294.41)	0.575
Final follow-up	271.11 (242.99–295.56)	225.28 (212.43–238.44)	226.47 (202.07–251.46)	0.022
*p*	0.066	0.014	0.038	
**Butter**				
Baseline	12.04 (5.61–18.46)	19.44 (13.92–24.97)	20.44 (12.23–28.65)	0.373
Final follow-up	11.85 (6.47–17.23)	14.58 (9.79–19.38)	15.88 (6.42–25.35)	0.608
*p*	0.981	0.077	0.071	
**HEI (score 0–24)**				
Baseline	15.11 (14.06–16.16)	15.59 (15.15–16.03)	14.91 (13.86–15.96)	0.394
Final follow-up	15.74 (14.69–16.80)	16.20 (15.77–16.63)	15.53 (14.48–16.58)	0.475
*p*	0.481	0.018	0.208	

All measures are expressed as mean of grams per week or milliliters per week with corresponding 95% CI in brackets. HEI: Healthy Eating Index.

*Kruskal–Wallis test in rows; Wilcoxon test in columns.

Patients who lost weight reported at baseline a greater consumption of potatoes and milk and lower consumption of breadsticks or crackers, eggs, aged cheese, snacks, and wine than their counterparts. This patient group increased oil consumption after chemotherapy: this finding contrasts with the other groups, who reported a reduction of this condiment at the final follow-up (p = 0.02). In addition, these patients reported greater reductions in sugar and beer consumption than the other groups, being the lowest among them at T2.

Conversely, patients who gained weight reported at baseline the greatest consumption of breadsticks, fish, and aged cheese, but the lowest intake of milk and sugar. Notably, this was the category in which sugar consumption did not statistically decrease but remained consistent or slightly increased after chemotherapy.

As reported in Table 5 the HEI increased from T0 to T2 *.*The change of kilocalories intake variation across the three weight categories (table S3) was consistent with the food habit variation across the three weight categories (stable, increase a d decrease) reported in Table 5.

As pointed out in Table S4 of supplementary materials, we observed in each weight categories a statistically significant reduction of employment while physical activity decreased but not significantly from baseline to final follow-up visit; however, no differences in terms of employment and physical activity were observed across the same weight categories.

## Discussion

Research on food preferences during chemotherapy is mostly anecdotal and scarcely measured quantitatively. The presented study is one of the few evaluators of dietary habit changes in EBC patients undergoing adjuvant chemotherapy^5^.

The main results show that EBC patients significantly reduced the consumption of pasta or rice, bread, breadsticks, red meat, fat and lean salami, cheese, milk, yogurt, added sugar, soft drinks, alcoholic beverages, and condiments, whereas they increased fruit intake. As a matter of fact, a significant increase in the HEI was observed after chemotherapy completion, suggesting an improvement in the quality of diet and adoption healthier dietary patterns.

Furthermore, as expected, a reduction in physical activity during and after chemotherapy was recorded; therefore, we believe that the change in eating habits towards a healthier and lower caloric diet could be the main reason why no weight gain in most patients after adjuvant chemotherapy was observed. These data are in contrast with several studies reporting weight gain in the majority of women diagnosed with breast cancer following adjuvant treatments^6–12^. As a matter of fact, a recent Australian survey found that two-thirds of the EBC patients interviewed reported an average weight gain of 9.07 kg within the first 12 months^31^. However, trials using predominantly modern and short term adjuvant chemotherapy regimens failed to show weight gain during treatment^33–34^.

Most commonly, patients modified their diet through a reduction in consumption of animal fat, read meat, processed meat, added sugar, milk and other dairy products, bread, cereals and through a rise in fruit consumption: these findings are partially consistent with those reported by other authors^35^.

Regarding the intake of energy and macronutrients, EBC patients reported a significantly lower total energy, fat, protein, and carbohydrate intake during and after treatment. A recently published paper found out that EBC patients reported at baseline mean energy, protein, fat and carbohydrate intake similar to women without cancer^36^, while during chemotherapy EBC patients reported a significantly lower intake of energy, fat, and proteins but not carbohydrates than their counterpart without cancer. Wayne et al. reported a small but significant decrease in energy and macronutrient intake after breast cancer diagnosis, including carbohydrates^22^.

These data, together with those reported in the present study, show that EBC patients nowadays are highly motivated and inclined to change eating habits towards a healthier direction early during adjuvant treatment, in order to prevent weight increase and potentially improve treatment efficacy^22,28,37,38^. Therefore, our results are in contrast with those showing adjuvant chemotherapy as an independent predictive factor of weight gain in EBC patients^6–12^.

Despite the general tendency of our patients to maintain stable weight, 16.6% of them gained and 13.2% lost weight: we observed that patients with higher body weight and BMI at baseline had a lower tendency to gain weight during and after adjuvant chemotherapy, while the opposite was true in patients with lower weight. These data are consistent with the results of several studies showing that higher BMI and body weight before breast cancer diagnosis were associated with less likelihood of gaining weight afterwards^39,40,41^. As secondary aim we investigated the potential relationship between food preferences and body weight variations before and after chemotherapy: our results pointed out differences between patients who gained and those who lost weight, particularly as regards to sugar and oil consumption, which decreased and increased respectively in the latter but not in the former group.

These data could be helpful for health professionals to develop tailored interventions that support EBC patients to handle or prevent treatment-related weight increase and that ultimately improve their quality of life and future health. In our study changes in eating habits already occurred at the first follow-up during chemotherapy and did not substantially modify at the end of treatment: this observation suggests that the dietician’s intervention should occur early and timely. As previously reported, EBC patients reduces consistently their physical activity^15,16,42^ and most of them did not work during chemotherapy^43,44^, which was confirmed in our series. No differences in terms of physical activity were found among the three weight categories and this is a rather surprising finding, considering the fundamental role of physical exercise in preventing weight gain.

The strengths of our study include the prospective design with data collection and dietary assessment in three consecutive time points carried out by a trained dietician.

There are also a few limitations of our study: the dietary assessment was not conducted with a validated food frequency questionnaire, mainly because few Italian food frequency questionnaire are validated and available^45^. However, the use of food photographs to quantify food consumption and portion sizes, although not validated, was adopted in other several studies^46^. Furthermore, no correction for multiple comparisons were made.

In conclusion, our prospective study shows that EBC patients tend to adopt “healthier dietary patterns”, in agreement with WCRF recommendations^28^, during and after adjuvant chemotherapy, leading to a non-change in body weight, despite reduction of physical activity. Eating habits changes in relation to body weight variations observed in the present study, although exploratory, could be useful for the implementation of personalized diets and life-style recommendations.

## Supplementary Information

Supplementary Information.

## References

[1] Jemal A, Center MM, DeSantis C, Ward EM (2010). Global patterns of cancer incidence and mortality rates and trends. Cancer Epidemiol. Biomark. Prev..

[2] Munoz D (2014). Effects of screening and systemic adjuvant therapy on ER-specific US breast cancer mortality. J. Natl. Cancer Inst..

[3] Runowicz CD (2016). American cancer society/American society of clinical oncology breast cancer survivorship care guideline. J. Clin. Oncol..

[4] Chlebowski RT, Aiello E, McTiernan A (2002). Weight loss in breast cancer patient management. J. Clin. Oncol..

[5] Vance V, Mourtzakis M, Mccargar L, Hanning R (2011). Weight gain in breast cancer survivors: prevalence, pattern and health consequences. Obes. Rev..

[6] Freedman RJ (2004). Weight and body composition changes during and after adjuvant chemotherapy in women with breast cancer. J. Clin. Endocrinol. Metab..

[7] Heideman WH, Russell NS, Gundy C, Rookus MA, Voskuil DW (2009). The frequency, magnitude and timing of post-diagnosis body weight gain in Dutch breast cancer survivors. Eur. J. Cancer.

[8] Goodwin PJ (1999). Adjuvant treatment and onset of menopause predict weight gain after breast cancer diagnosis. J. Clin. Oncol..

[9] Irwin ML (2005). Changes in body fat and weight after a breast cancer diagnosis: Influence of demographic, prognostic, and lifestyle factors. J. Clin. Oncol..

[10] Demark-Wahnefried, W., Rimer, B. K. & Winer, E. P. Weight gain in women diagnosed with breast cancer. *J. Am. Diet. Assoc.* **97**, 519–26, 529; quiz 527–8 (1997). 10.1016/s0002-8223(97)00133-8 9145091

[11] Demark-Wahnefried W, Winer EP, Rimer BK (1993). Why women gain weight with adjuvant chemotherapy for breast cancer. J. Clin. Oncol..

[12] Makari-Judson G, Braun B, JosephJerry D, Mertens WC (2014). Weight gain following breast cancer diagnosis: Implication and proposed mechanisms. World J. Clin. Oncol..

[13] Berg MMGA (2017). Weight change during chemotherapy in breast cancer patients: a meta-analysis. BMC Cancer.

[14] Thomson ZO, Reeves MM (2017). Can weight gain be prevented in women receiving treatment for breast cancer? A systematic review of intervention studies. Obes. Rev..

[15] Pekmezi DW, Demark-Wahnefried W (2011). Updated evidence in support of diet and exercise interventions in cancer survivors. Acta Oncol..

[16] Irwin ML (2003). Physical activity levels before and after a diagnosis of breast carcinoma: the health, eating, activity, and lifestyle (HEAL) study. Cancer.

[17] Demark-Wahnefried W (2001). Changes in weight, body composition, and factors influencing energy balance among premenopausal breast cancer patients receiving adjuvant chemotherapy. J. Clin. Oncol..

[18] Harvie MN, Campbell IT, Baildam A, Howell A (2004). Energy balance in early breast cancer patients receiving adjuvant chemotherapy. Breast Cancer Res. Treat..

[19] Grindel CG, Cahill CA, Walker M (1989). Food intake of women with breast cancer during their first six month of chemotherapy. Oncol. Nurs. Forum.

[20] Kosaka Y (2016). Phase II randomized, controlled trial of 1 day versus 3 days of dexamethasone combined with palonosetron and aprepitant to prevent nausea and vomiting in Japanese breast cancer patients receiving anthracycline-based chemotherapy. Support. Care Cancer.

[21] Serra MC, Goldberg AP, Ryan AS (2016). Increased depression and metabolic risk in postmenopausal breast cancer survivors. Diabetol. Metab. Syndr..

[22] Wayne SJ (2004). Changes in dietary intake after diagnosis of breast cancer. J. Am. Diet. Assoc..

[23] Cardoso F (2019). Early breast cancer: ESMO Clinical Practice Guidelines for diagnosis, treatment and follow-up. Ann. Oncol..

[24] Associazione Italiana di Oncologia Medica AIOM. Linee guida Neoplasie della Mammella. 311 (2019).

[25] Willett WC (1998). Nutritional Epidemiology.

[26] Fantuzzi, F., Chiuchiù, M. P. & Bedogni, G. Atlante fotografico delle porzioni degli alimenti. *Milano. Ist. Scotti Bassani* (2005).

[27] Società italiana di nutrizione umana. *LARN. Livelli di assunzione di riferimento di nutrienti ed energia per la popolazione italiana*. (SICS, 2014).

[28] World Cancer Research Fund/American Institute for Cancer Research. *Diet, Nutrition, Physical Activity and Cancer: a Global Perspective* (2018).

[29] Castro-Quezada I, Román-Viñas B, Serra-Majem L (2014). The Mediterranean diet and nutritional adequacy: a review. Nutrients.

[30] CREA. *Linee guida per la sana alimentazone (Revisione 2018)*. www.crea.gov.it (2019).

[31] Ee C, Cave AE, Naidoo D, Bilinski K, Boyages J (2020). Weight before and after a diagnosis of breast cancer or ductal carcinoma in situ: a national Australian survey. BMC Cancer.

[32] Klein S (2004). Clinical implications of obesity with specific focus on cardiovascular disease: a statement for professionals from the American Heart Association Council on Nutrition, Physical Activity, and Metabolism. Circulation.

[33] Freeman RJ (2004). Weight and body composition changes during and after adjuvant chemotherapy in women with breast cancer. J. Clin. Endocrinol. Metab..

[34] Kutynec CL, McCargar L, Barr SI, Hislop TG (1999). Energy balance in women with breast cancer during adjuvant treatment. J. Am. Diet. Assoc..

[35] Salminen E, Bishop M, Poussa T, Drummond R, Salminen S (2004). Dietary attitudes and changes as well as use of supplements and complementary therapies by Australian and Finnish women following the diagnosis of breast cancer. Eur. J. Clin. Nutr..

[36] de Vries YC (2017). Differences in dietary intake during chemotherapy in breast cancer patients compared to women without cancer. Support. Care Cancer.

[37] Salminen EK, Lagström HK, Heikkilä SP, Salminen SJ (2000). Does breast cancer change patients’ dietary habits?. Eur. J. Clin. Nutr..

[38] Thomson CA (2002). Increased fruit, vegetable and fiber intake and lower fat intake reported among women previously treated for invasive breast cancer. J. Am. Diet. Assoc..

[39] Makari-Judson G, Judson CH, Mertens WC (2007). Longitudinal patterns of weight gain after breast cancer diagnosis: observations beyond the first year. Breast J..

[40] Gu K (2010). Weight change patterns among breast cancer survivors: Results from the Shanghai breast cancer survival study. Cancer Causes Control.

[41] Nissen MJ, Shapiro A, Swenson K (2011). Changes in weight and body composition in women receiving chemotherapy for breast cancer. Clin. Breast Cancer.

[42] Fassier P (2016). Variations of physical activity and sedentary behavior between before and after cancer diagnosis: results from the prospective population-based NutriNet-Santé cohort. Medicine (United States).

[43] Grunfeld EA, Cooper AF (2012). A longitudinal qualitative study of the experience of working following treatment for gynaecological cancer. Psychooncology..

[44] Short PF, Vasey JJ, Tunceli K (2005). Employment pathways in a large cohort of adult cancer survivors. Cancer.

[45] Franceschi S (1995). Reproducibility of an Italian food frequency questionnaire for cancer studies. Results for specific nutrients. Ann. Epidemiol..

[46] Nelson M, Atkinson M, Darbyshire S (1996). Food photography II: use of food photographs for estimating portion size and the nutrient content of meals. Br. J. Nutr..

