# Supplementary material for: Changes in eating habits and food preferences in breast cancer patients undergoing adjuvant chemotherapy
Source: Sci Rep. 2021 Jun 21;11:12975. doi: 10.1038/s41598-021-92138-7 (PMC8217237; doi:10.1038/s41598-021-92138-7)
Supplement: Supplementary file 1 — Supplementary Information. [file 41598_2021_92138_MOESM1_ESM.doc]

**Changes in eating habits and food preferences in breast cancer patients undergoing adjuvant chemotherapy**

Rebecca Pedersini^1,2^, Pierluigi di Mauro^1^, Sara Bosio^2^, Barbara Zanini^3^, Alessandra Zanini^1^, Vito Amoroso^1^, Antonella Turla^1^, Lucia Vassalli^1,2^, Mara Ardine^1^, Sara Monteverdi^1^, Manuel Zamparini^1^, Cristina Gurizzan^1^, Deborah Cosentini^1^, Chiara Ricci^4^, Edda Lucia Simoncini^2^, Alfredo Berruti^1^

**Supplementary Table S1**. Score assignment for the Healthy Eating Index (HEI) in the CHANGE study.

| *Item* | *Frequency of Consumption** | *Score* |
| --- | --- | --- |
| Fresh Fruit | - Every day - 5-6/weekly - 3-4/weekly or less | - 2 - 1 - 0 |
| Vegetables | - Every day - 5-6/weekly - 3-4/weekly or less | - 2 - 1 - 0 |
| Potatoes | - 1-2/weekly or less - 3-4/weekly or more | - 1 - 0 |
| Poultry | - 1-2/weekly or less - 3-4/weekly or more | - 1 - 0 |
| Red meat | - 1-2/monthly or less - 1-2/weekly - 3-4/weekly or more | - 2 - 1 - 0 |
| Fish | - 1-4/weekly - 1-2/monthly or 4-5/weekly - Never or everyday | - 2 - 1 - 0 |
| Lean salami | - 1-2/monthly or less - 1-2/weekly or more | - 1 - 0 |
| Eggs | - 1-4/weekly - 1-2/monthly or 4-5/weekly - Never or everyday | - 2 - 1 - 0 |
| Fresh cheese | - 1-4/weekly - 1-2/monthly or 4-5/weekly - Never or everyday | - 2 - 1 - 0 |
| Legumes | - 3-4/weekly or more - 1-2/weekly - 1-2/monthly or less | - 2 - 1 - 0 |
| Snacks | - 1-2/weekly or less - 3-4/weekly or more | - 1 - 0 |
| Soft drinks | - Never - 1-2/monthly or 1-2/weekly - 3-4/weekly or more | - 2 - 1 - 0 |
| Oil | - Every day - 5-6/weekly - 3-4/weekly or less | - 2 - 1 - 0 |
| Nuts and seeds | - 5-6/weekly or more - 1-4/weekly - 1-2/monthly or less | - 2 - 1 - 0 |

**Score range: 0-24**

* Frequency of consumption refers to an Italian standard serving size for every food item (according to LARN 2014)^31^.

**Supplementary Table S2**. Weight and BMI variations across the three weight categories (decreased/stable/increased)

|  | **Decreased weight** | **Stable weight** | **Increased weight** | **p*** |
| --- | --- | --- | --- | --- |
| **Weight** (kg)  First follow-up  Final follow-up | 69.27 (64.23-74.31)  63.86 (59.35-68.37) | 65.07 (63-67)  65.13 (63-67) | 60.22 (56.7-63.6)  66.69 (62.4-70.9) | 0.014  0.558 |
| **Weight difference** (kg) | - 5.41 | - 0.06 | + 6.47 |  |
| **p*** | < 0.001 | 0.670 | < 0.001 |  |
| **BMI** (kg/m^2^)  First follow-up  Final follow-up | 26.66 (24.72-28.60)  24.71 (22.94-26.48) | 24.88 (24.08-25.68)  24.76 (23.9-25.6) | 22.87 (21.7-23.9)  25.11 (23.7-26.43) | 0.008  0.718 |
| **BMI difference** (kg/m^2^) | - 1.95 | - 0.12 | + 2.24 |  |
| **p*** | < 0.001 | 0.477 | < 0.001 |  |

Kg: kilogram; BMI: body max index.

* Friedman’s test in rows and Wilcoxon test in columns.

**Supplementary Table S3**. Kilocalories intake variation across the three weight categories.

| **Food or beverage** | **Decreased weight** | **Stable weight** | **Increased weight** | **p** |
| --- | --- | --- | --- | --- |
| **Fruit**  Baseline  Final follow-up | 1083.44 (810.47-1356.41)  1015.38 (852.74-1178.03) | 851.37 (756.18-946.56)  1129.80 (1020.86-1238.75) | 938.20 (739.52-1136.88)  1053.86 (875.74-1231.97) | 0.276  0.790 |
| **p** | 0.754 | < 0.001 | 0.222 |  |
| **Vegetables**  Baseline  Final follow-up | 427.85 (355.09-500.62)  477.70 (405.41-550.00) | 499.16 (460.67-537.65)  491.51(455.71-527.31) | 543.26 (455.02-631.51)  547.09 (462.34-631.84) | 0.202  0.453 |
| **p** | 0.236 | 0.942 | 0.988 |  |
| **Bread**  Baseline  Final follow-up | 1376.19 (956.81-1795.56)  895.70 (622.06-1169.34) | 1179.51 (1038.68-1320.33)  893.10 (781.94-1004.27) | 1107.41 (832.09-1382.73)  912.50 (690.54-1134.46) | 0.661  0.977 |
| **p** | 0.024 | < 0.001 | 0.073 |  |
| **Breadsticks/crackers**  Baseline  Final follow-up | 60 (22.22-97.78)  21.11 (5.83-36.40) | 108.33 (83.91-132.76)  56.04 (39.63-72.45) | 125.74 (75.75-175.72)  51.62 (27.61-75.63) | 0.072  0.263 |
| **p** | 0.040 | < 0.001 | 0.004 |  |
| **Pasta**  Baseline  Final follow-up | 358.15 (282.33-433.96)  357.78 (276.46-439.10) | 400.35 (365.85-434.84)  368.26 (337.42-399.10) | 373.24 (298.35-448.12)  342.94 (269.71-416.17) | 0.552  0.787 |
| **p** | 0.896 | 0.113 | 0.346 |  |
| **Potatoes**  Baseline  Final follow-up | 281.48 (200.03-362.94)  225.19 (174.74-275.64) | 195.83 (168.17-223.50)  183.61 (156.49-229.73) | 201.17 (150.04-252.31)  203.53 (130.53-276.53) | 0.088  0.232 |
| **p** | 0.209 | 0.529 | 0.852 |  |
| **Fish**  Baseline  Final follow-up | 285.67 (222.24-349.09)  282.74 (220.32-345.17) | 244.57 (217.83-271.31)  262.78 (234.20-291.36) | 332.68 (243.29-422.06)  341.18 (244.53-437.82) | 0.123  0.360 |
| **p** | 0.893 | 0.204 | 0.866 |  |
| **Read meat**  Baseline  Final follow-up | 146.30 (108.72-183.88)  113.89 (77.05-150.72) | 128.13 (109.20-147.05)  102.95 (88.18-117.73) | 140.44 (98.47-182.41)  85.29 (64.91-105.68) | 0.255  0.672 |
| **p** | 0.179 | 0.012 | 0.040 |  |
| **White meat**  Baseline  Final follow-up | 263.89 (191.61-336.16)  225 (186.31-263.69) | 238.19 (210.88-265.51)  232.64 (207.88-257.40) | 208.82 (168.54-249.11)  203.68 (158.78-248.57) | 0.686  0.654 |
| **p** | 0.254 | 0.812 | 0.797 |  |
| **Lean salami**  Baseline  Final follow-up | 313.67 (195.92-431.41)  214.48 (152.42-276.54) | 287.54 (240.86-334.22)  187.76 (154.31-221.22) | 309.26 (216.23-402.30)  215.03 (130.42-299.64) | 0.508  0.417 |
| **p** | 0.274 | < 0.002 | 0.055 |  |
| **Fat salami**  Baseline  Final follow-up | 26.89 (9.93-43.84)  15.89 (4.23-27.55) | 34.48 (26.79-42.17)  16.14 (10.23-22.04) | 56.29 (23.05-89.53)  33.97 (7.97-59.97) | 0.477  0.333 |
| **p** | 0.148 | < 0.001 | 0.067 |  |
| **Eggs**  Baseline  Final follow-up | 113.78 (74.16-153.40)  110.22 (75.41-145.04) | 158.44 (134.75-182.14)  142.89 (124.56-161.22) | 151.53 (111.29-191.77)  143.06 (114.59-171.52) | 0.146  0.266 |
| **p** | 0.917 | 0.185 | 0.875 |  |
| **Milk**  Baseline  Final follow-up | 260.19 (136.25-384.12)  230.22 (130.34-330.10) | 211.26 (159.84-262.69)  167.71 (124.98-210.44) | 116.03 (58.56-173.50)  130.47 (64.48-196.46) | 0.273  0.269 |
| **p** | 0.469 | 0.078 | 0.652 |  |
| **Fresh cheese**  Baseline  Final follow-up | 553.19 (369.33-737.04)  461.89 (316.97-606.81) | 499.26 (420.60-577.92)  403.96 (338.93-468.99) | 422.56 (269.97-575.15)  337.03 (242.15- 431.91) | 0.485  0.451 |
| **p** | 0.206 | 0.035 | 0.477 |  |
| **Aged cheese**  Baseline  Final follow-up | 297.30 (174.60-419.99)  277.52 (157.94-397.10) | 393.94 (321.88-465.01)  302.94 (250.50-355.39) | 477.71 (346.51-608.90)  317.35 (204.58-430.13) | 0.073  0.966 |
| **p** | 1 | 0.029 | 0.070 |  |
| **Legumes**  Baseline  Final follow-up | 243.63 (155.32-337.94)  186.33 (116.34-256.33) | 258.82 (213.95-303.69)  250.94 (206.73-295.15) | 215.29 (147.48-283.10)  269.41 (181.55-357.27) | 0.835  0.538 |
| **p** | 0.378 | 0.735 | 0.404 |  |
| **Sugar**  Baseline  Final follow-up | 236.26 (123.53-348.98)  144.48 (56.53-232.43) | 338.24 (275.43-401.04)  236.67(186.84-286.50) | 162.29 (82.13-242.46)  198.00 (99.34-296.66) | 0.011  0.109 |
| **p** | 0.018 | < 0.001 | 0.400 |  |
| **Biscuits**  Baseline  Final follow-up | 552.96 (333.36-772.57)  511.78 (316.22-707.33) | 609.99(505.32-713.65)  497.90 (409.42-286.38) | 814.68 (537.60-1091.75)  637.21 (405.05-869.36) | 0.361  0.586 |
| **p** | 0.945 | 0.081 | 0.113 |  |
| **Snacks**  Baseline  Final follow-up | 492.22 (193.50-790.94)  653.30 (326.07-980.53) | 749.57(563.72-935.41)  589.96 (438.89-741.03) | 683.15 (431.97-934.32)  566.21 (305.50-826.91) | 0.178  0.630 |
| **p** | 0.541 | 0.153 | 0.152 |  |
| **Wine**  Baseline  Final follow-up | 122.85 (14.93-230.77)  81.07 (4.59-157.55) | 202.90 (149.04-256.77)  98.10 (65.11-131.08) | 197.62 (100.31-294.93)  93.26 (32.81-153.72) | 0.136  0.330 |
| **p** | 0.182 | < 0.0001 | 0.005 |  |
| **Beer**  Baseline  Final follow-up | 33.85(14.58-82.29)  2.48 (0.62-7.58) | 35.15 (21.35-48.94)  19.19 (9.07-29.31) | 33.47 (11.02-55.92)  19.03 (5.06-33.00) | 0.278  0.095 |
| **p** | 0.141 | 0.002 | 0.066 |  |
| **Oil**  Baseline  Final follow-up | 2100 (1790.88-2409.12)  2440 (2188.57-2691.43) | 2207.19 (2079.72-2374.76)  2027.50 (1916.62-2138.38) | 2334.71 (1998.80-2670.61)  2038.24 (1812.73-2263.74) | 0.575  0.022 |
| **p** | 0.066 | 0.014 | 0.038 |  |
| **Butter**  Baseline  Final follow-up | 91.26 (42.55-139.97)  89.74 (49.04-130.44) | 147.47 (105.59-189.34)  110.60 (74.25-146.96) | 155.03 (92.78-217.28)  120.47 (48.75-192.19) | 0.373  0.608 |
| **p** | 0.905 | 0.067 | 0.064 |  |

All measures are expressed as mean of kilocalories per week with corresponding 95% CI in brackets.

* Kruskal-Wallis test in rows; Wilcoxon test in columns.

**Supplementary Table S4**. Physical activity and employment variations across the three weight categories.

|  | **Decreased weight** | **Stable weight** | **Increased weight** | **p** |
| --- | --- | --- | --- | --- |
| Physical activity  Baseline, N (%)  Final control, N (%) | 15 (55.6)  12 (44.4) | 74 (51.4)  62 (43.1) | 19 (55.9)  15 (44.1) | 0.850  0.987 |
| p | 0.366 | 0.077 | 0.248 |  |
| Employment  Baseline, N (%)  Final control, N (%) | 18 (66.7)  7 (25.9) | 106 (73.6)  63 (43.8) | 28 (82.4)  19 (55.9) | 0.367  0.063 |
| p | 0.002 | < 0.001 | 0.013 |  |

Numbers and (percentages in brackets) of patients that practice physical activity or are employed before starting chemotherapy and after the end of treatment.

* Chi-squared test in rows; Wilcoxon test in columns.

**BASELINE QUESTIONNAIRE**

**Interview date:** ____/____/_________

| **Name and Surname** |  | |
| --- | --- | --- |
| **Date of birth** |  | **Age:** ___________ years old |
| **Telephone number** |  | |
| **Employment** |  | |
| **Physical Activity** | - Inactivity  Moderate  Vigorous   Number of minutes/week:………… | |
| **Menopausal Status** | - Premenopausal status  Postmenopausal status - Post menopausa - Post menopausa | |
| **Other current diseases** |  | |
| **Current drugs** |  | |
| **Chemotherapy regimen** |  | |

| **FOOD OR DRINK** | **SERVING SIZE** | | **FREQUENCY OF CONSUMPTION** | | | | | | | | | |
| --- | --- | --- | --- | --- | --- | --- | --- | --- | --- | --- | --- | --- |
|  | **Portion** | | **Never, or less than once per month** | **1-3 per month** | **1 per week** | **2-4 per week** | **5-6 per week** | **1 per day** | **2-3 per day** | **4-5 per day** | **6+ per day** |  |
|  | | | | | | | | | | | |  |
| Fruit |  |  | |  |  |  |  |  |  |  |  |  |
| Vegetables (potatoes are not considered vegetables) |  |  | |  |  |  |  |  |  |  |  |  |
|  | | | | | | | | | | | |  |
| Pasta or rice |  |  | |  |  |  |  |  |  |  |  |  |
| Bread |  |  | |  |  |  |  |  |  |  |  |  |
| Potatoes |  |  | |  |  |  |  |  |  |  |  |  |
| Breadsticks or crackers |  |  | |  |  |  |  |  |  |  |  |  |
|  | | | | | | | | | | | |  |
| White meat or poultry |  |  | |  |  |  |  |  |  |  |  |  |
| Red meat |  |  | |  |  |  |  |  |  |  |  |  |
| Fish |  |  | |  |  |  |  |  |  |  |  |  |
| Lean salami |  |  | |  |  |  |  |  |  |  |  |  |
| Fat salami |  |  | |  |  |  |  |  |  |  |  |  |
| Eggs |  |  | |  |  |  |  |  |  |  |  |  |
| Fresh cheese |  |  | |  |  |  |  |  |  |  |  |  |
| Aged cheese |  |  | |  |  |  |  |  |  |  |  |  |
| Legumes |  |  | |  |  |  |  |  |  |  |  |  |
| Milk |  |  | |  |  |  |  |  |  |  |  |  |
| Yogurt |  |  | |  |  |  |  |  |  |  |  |  |
|  | | | | | | | | | | | |  |
| Sugar |  |  | |  |  |  |  |  |  |  |  |  |
| Ice creams |  |  | |  |  |  |  |  |  |  |  |  |
| Snacks |  |  | |  |  |  |  |  |  |  |  |  |
| Soft drinks |  |  | |  |  |  |  |  |  |  |  |  |
| Wine |  |  | |  |  |  |  |  |  |  |  |  |
| Beer |  |  | |  |  |  |  |  |  |  |  |  |
| Schnapps |  |  | |  |  |  |  |  |  |  |  |  |
| Oil |  |  | |  |  |  |  |  |  |  |  |  |
| Butter |  |  | |  |  |  |  |  |  |  |  |  |
| Nuts and seeds |  |  | |  |  |  |  |  |  |  |  |  |

**FOLLOW-UP VISIT**

**Interview date:** ____/____/_________

| **Name and Surname** |  |
| --- | --- |
| **Current weight** (Kg) |  |
| **Employment** |  present  not present |
| **Physical Activity** | - Inactivity  Moderate  Vigorous   Number of minutes/week:………… |

| **FOOD OR DRINK** | **SERVING SIZE** | **FREQUENCY OF CONSUMPTION** | | | | | | | | | |
| --- | --- | --- | --- | --- | --- | --- | --- | --- | --- | --- | --- |
|  | **Portion** | **Never, or less than once per month** | **1-3 per month** | **1 per week** | **2-4 per week** | **5-6 per week** | **1 per day** | **2-3 per day** | **4-5 per day** | **6+ per day** |  |
|  | | | | | | | | | | |  |
| Fruit |  |  |  |  |  |  |  |  |  |  |  |
| Vegetables (potatoes are not considered vegetables) |  |  |  |  |  |  |  |  |  |  |  |
|  | | | | | | | | | | |  |
| Pasta or rice |  |  |  |  |  |  |  |  |  |  |  |
| Bread |  |  |  |  |  |  |  |  |  |  |  |
| Potatoes |  |  |  |  |  |  |  |  |  |  |  |
| Breadsticks or crackers |  |  |  |  |  |  |  |  |  |  |  |
|  | | | | | | | | | | |  |
| White meat or poultry |  |  |  |  |  |  |  |  |  |  |  |
| Red meat |  |  |  |  |  |  |  |  |  |  |  |
| Fish |  |  |  |  |  |  |  |  |  |  |  |
| Lean salami |  |  |  |  |  |  |  |  |  |  |  |
| Fat salami |  |  |  |  |  |  |  |  |  |  |  |
| Eggs |  |  |  |  |  |  |  |  |  |  |  |
| Fresh cheese |  |  |  |  |  |  |  |  |  |  |  |
| Aged cheese |  |  |  |  |  |  |  |  |  |  |  |
| Legumes |  |  |  |  |  |  |  |  |  |  |  |
| Milk |  |  |  |  |  |  |  |  |  |  |  |
| Yogurt |  |  |  |  |  |  |  |  |  |  |  |
|  | | | | | | | | | | |  |
| Sugar |  |  |  |  |  |  |  |  |  |  |  |
| Ice creams |  |  |  |  |  |  |  |  |  |  |  |
| Snacks |  |  |  |  |  |  |  |  |  |  |  |
| Soft drinks |  |  |  |  |  |  |  |  |  |  |  |
| Wine |  |  |  |  |  |  |  |  |  |  |  |
| Beer |  |  |  |  |  |  |  |  |  |  |  |
| Schnapps |  |  |  |  |  |  |  |  |  |  |  |
| Oil |  |  |  |  |  |  |  |  |  |  |  |
| Butter |  |  |  |  |  |  |  |  |  |  |  |
| Nuts and seeds |  |  |  |  |  |  |  |  |  |  |  |

**Figure 1.** Timeline of the dietary assessment.

Baseline (T0), before chemotherapy

First follow-up (T1)

2-3 months after starting chemotherapy

Final follow-up (T2)

One week after ending chemotherapy
